# Matrix Metalloproteinase-9 Expression Is Associated with the Absence of Response to Neoadjuvant Chemotherapy in Triple-Negative Breast Cancer Patients

**DOI:** 10.3390/ijms241411297

**Published:** 2023-07-11

**Authors:** Marylène Lejeune, Laia Reverté, Noèlia Gallardo, Esther Sauras, Ramon Bosch, Daniel Mata, Albert Roso, Anna Petit, Vicente Peg, Francisco Riu, Joan García-Fontgivell, Fernanda Relea, Begoña Vieites, Luis de la Cruz-Merino, Meritxell Arenas, Valeri Rodriguez, Juana Galera, Anna Korzynska, Benoît Plancoulaine, Tomás Álvaro, Carlos López

**Affiliations:** 1 Oncological Pathology and Bioinformatics Research Group, Molecular Biology and Research Section, Pathology Department, Hospital de Tortosa Verge de la Cinta, Institut d’Investigació Sanitària Pere Virgili (IISPV), Universitat Rovira i Virgili (URV), Esplanetes, 14, 43500 Tortosa, Spain; mlejeune.ebre.ics@gencat.cat (M.L.); noelia.gabo@gmail.com (N.G.); esauras.ebre.ics@gencat.cat (E.S.); rbosch.ebre.ics@gencat.cat (R.B.); dmata.ebre.ics@gencat.cat (D.M.); talvaro.ebre.ics@gencat.cat (T.Á.); 2 Clinical Studies Unit, Hospital de Tortosa Verge de la Cinta, Carretera Esplanetes, 14, 43500 Tortosa, Spain; 3 Institut Universitari d’Investigació en Atenció Primària Jordi Gol (IDIAP Jordi Gol), Gran Via Corts Catalanes, 587, 08007 Barcelona, Spain; aroso@idiapjgol.org; 4 Pathology Department, Hospital Universitari de Bellvitge, 08907 Barcelona, Spain; apetit@bellvitgehospital.cat; 5 Pathology Department, Hospital Universitari de Vall Hebron, 08035 Barcelona, Spain; vpeg@vhebron.net; 6 Pathology Department, Hospital Universitari Sant Joan de Reus, 43204 Reus, Spain; francesc.riu@salutsantjoan.cat; 7 Pathology Department, Hospital Universitari Joan XXIII, Institut d’Investigació Sanitària Pere Virgili (IISPV), 43005 Tarragona, Spain; jgarciaf.hj23.ics@gencat.cat; 8 Pathology Department, Hospital General de Ciudad Real, 13005 Ciudad Real, Spain; fernandar@sescam.jccm.es; 9 Pathology Department, Hospital Universitario Virgen del Rocío, 41013 Seville, Spain; mb.vieites.sspa@juntadeandalucia.es; 10 Oncology Department, Hospital Universitario Virgen Macarena, 41009 Seville, Spain; luis.cruz.sspa@juntadeandalucia.es; 11 Department of Radiation Oncology, Hospital Universitari Sant Joan de Reus, Institut d’Investigació Sanitària Pere Virgili (IISPV), Universitat Rovira i Virgili, 43204 Tarragona, Spain; meritxell.arenas@urv.cat; 12 Oncology Department, Hospital de Tortosa Verge de la Cinta, Institut d’Investigació Sanitària Pere Virgili (IISPV), 43500 Tortosa, Spain; dra.rodriguezg@gmail.com; 13 Gynaecology Department, Hospital Universitari Joan XXIII, Institut d’Investigació Sanitària Pere Virgili (IISPV), 43005 Tarragona, Spain; jgalor@tinet.org; 14 Laboratory of Processing and Analysis of Microscopic Images, Nalecz Institute of Biocybernetics and Biomedical Engineering, Polish Academy of Sciences, 02-109 Warsaw, Poland; akorzynska@ibib.waw.pl; 15 ANTICIPE, INSERM, François Baclesse Comprehensive Cancer Center, University Caen Normandy, 14000 Caen, France; benoit.plancoulaine@orange.fr

**Keywords:** triple-negative breast cancer, neoadjuvant chemotherapy, tumor microenvironment, biomarkers, pathological response

## Abstract

Triple-negative breast cancer (TNBC) is particularly challenging due to the weak or absent response to therapeutics and its poor prognosis. The effectiveness of neoadjuvant chemotherapy (NAC) response is strongly influenced by changes in elements of the tumor microenvironment (TME). This work aimed to characterize the residual TME composition in 96 TNBC patients using immunohistochemistry and in situ hybridization techniques and evaluate its prognostic implications for partial responders vs. non-responders. Compared with non-responders, partial responders containing higher levels of CD83+ mature dendritic cells, FOXP3+ regulatory T cells, and IL-15 expression but lower CD138+ cell concentration exhibited better OS and RFS. However, along with tumor diameter and positive nodal status at diagnosis, matrix metalloproteinase-9 (MMP-9) expression in the residual TME was identified as an independent factor associated with the impaired response to NAC. This study yields new insights into the key components of the residual tumor bed, such as MMP-9, which is strictly associated with the lack of a pathological response to NAC. This knowledge might help early identification of TNBC patients less likely to respond to NAC and allow the establishment of new therapeutic targets.

## 1. Introduction

Triple-negative breast cancer (TNBC) represents 12–17% of all breast cancers (BCs) [1]. Its tumors are notable for their aggressive nature and are usually associated with high distal recurrence and poor survival rates [2]. The diverse molecular fingerprints of TNBCs have hindered the identification of predictive biomarkers of prognosis and response to therapeutics that are suitable for all BC subtypes. Moreover, despite progress towards this goal, clinical trial validation and standardized chemotherapy regimens remain poorly established [3]. Current treatments for early-stage or locally advanced TNBC consist of pre-operative neoadjuvant chemotherapy (NAC), which offers better chances for breast-conserving surgery and provides information about tumor behavior for future therapies and usually prolonged long-term outcomes [4]. Although 30–60% of TNBC patients achieve a pathological complete response (pCR) following NAC, those unable to develop it exhibit residual disease, which leads to a higher risk of metastatic recurrence and lower overall survival (OS) in the initial years after diagnosis [5,6]. In this regard, the pCR is a well-established surrogate marker of NAC effectiveness that is particularly useful in TNBC and HER2-positive subtypes [7]. Thus, pinpointing biomarkers in this setting becomes crucial for predicting the response to NAC, facilitating improved clinical decision-making. In this context, distinct components of the tumor microenvironment (TME) influence disease progression and the effectiveness of therapeutics [8]. Similarly, NAC is known to modify the TME content involved in the immune response, modulating antitumor response and even affecting the quality of the response to treatment [9]. In BC, high intratumoral immune infiltration dominated by T cells has been described as a predictive factor of favorable response to NAC [10]. Great efforts have been made in several studies to characterize the immune residual TME profile in TNBC patients, which have yielded promising biomarkers predicting the response to NAC, thereby allowing for better patient selection and personalized treatment [11]. However, the diverse prognostic implications of such TME elements depending on the type of response to NAC needs further investigation. In pursuing this challenge, our previous study revealed the potential role of elevated levels of lymphocytes (CD4 and FOXP3) and dendritic cells (CD21, CD1a and CD83) in the TME as useful markers to distinguish the TNBC patients with the most favorable outcomes despite not having attained pCR. In addition to cellular markers, we identified the lack of CXCL13 expression and the expression of MUC1 in the residual tumor as genetic risk factors strongly associated with higher recurrence and lower survival rates [12]. Given the proven relevance of residual TME as a prognostic factor, the present study compares the residual tumor composition between TNBC patients exhibiting a pathological partial response (“partial responders”) and those without a pathological response (“non-responders”) after NAC and explores the association of the selected markers with the type of response post-NAC.

## 2. Materials and Methods

### 2.1. Cohort Study Design

This retrospective cohort study involved 96 TNBC patients from a previous study [12] who were diagnosed with invasive breast carcinoma of no special type (NST) between 2008 and 2013 and followed up for as long as 5 years from the date of surgery. The biopsies of TNBC patients were obtained after NAC from cancer biobanks or tumor banks of the pathology departments of the participating centers (Hospital de Tortosa Verge de la Cinta, Hospital Universitari de Bellvitge, Hospital Universitari de Vall Hebron, Hospital Universitari Sant Joan de Reus, Hospital Universitari Joan XXIII, Hospital Universitario Virgen Macarena, Hospital General de Ciudad Real, Hospital Universitario Virgen del Rocío and Hospital Universitario Virgen Macarena). All participants provided written informed consent to participate in the study.

The cohort was divided into partial and non-responders depending on the pathological response to NAC, assessed in the surgical specimen obtained after the completion of therapy. The pathological response was determined with respect to Miller-Payne [13] and residual cancer burden (RCB) [14] grading systems. The comparative study of the partial responder and non-responder patient groups was based on the data collected in the previous study (demographic and clinico-pathological variables, the cellular markers content determined by immunohistochemistry (IHC) and the mRNA expression of cytokines and interleukins determined by chromogenic in situ hybridization (ISH)).

### 2.2. Tissue Microarray Construction and IHC & ISH Techniques

Two representative 2 mm diameter cylinders from the residual tumor biopsy site were extracted from the surgical specimen removed post NAC in each case of the study and transferred to a paraffin mold with the Arraymold tissue microarray (TMA) tool.

Afterwards, sections of 2 µm were made from each TMA to perform the IHC and ISH techniques. The IHC staining was performed by the ENDVISION FLEX™ (Santa Clara, CA, USA) method using diaminobenzidine chromogen (DAB) as the substrate, following the supplier’s instructions and laboratory protocol. The ISH assays were performed by Sophistolab (Sophilstolab AG, Muttenz, Switzerland) using the ViewRNA QuantiGene^®^ kit (Affymetrix, Inc., Santa Clara, CA, USA). The signal was detected using Fast Red chromogen substrate, and the presence of positive staining of mRNA was visualized by the presence of red dots. The antibodies’ sources and dilutions and the probes used in this study are listed in the supplementary material of the previous publication [12]. IHC- and ISH-stained slides of each TMA were digitized with an Aperio ScanScope CS slide scanner at 20X/NA 0.75 to produce LZW lossless compression profiles in SVS format.

### 2.3. Digital Image Analysis

Each cylinder of the scanned TMAs was extracted with specific automated algorithms as previously described [15] and saved as an individual image in TIFF format. The digital images obtained from the IHC-stained markers were evaluated by an automated digital image analysis procedure previously established [16] using FIJI software [17], which quantifies the percentage of positive-stained areas of each marker in relation to the whole area of the cylinder (in pixels), which is expressed as the mean percentages of the positive pixels of the two cylinders studied in each patient.

The digital images of stained IHS mRNA markers were evaluated manually by two specialized pathologists and classified into positive or negative mRNA expression based on the previously established cut-off value of 10%. Accordingly, negative mRNA expression was assigned to cylinders without staining or with fewer than 10% red dots and positive mRNA expression to cylinders with ≥10% red dots, choosing in each patient the highest value of the two cylinders studied.

### 2.4. Statistical Analysis

Following normality assessment using the Kolmogorov–Smirnov test, differences in the median concentrations of the IHC-stained markers between partial and non-responders were determined using unpaired samples *t*-tests for normally distributed data or Mann–Whitney U tests for non-normally distributed data. As appropriate, the chi-square or Fisher exact test was used to evaluate differences in the percentage of patients expressing mRNA of ISH-stained markers between partial and non-responders.

The Kaplan–Meier (K–M) method was used to estimate the OS and relapse-free survival (RFS) among all patients for each marker studied (determined by IHC and ISH). Superimposed K–M curves were derived for each type of response to NAC, stratified by the concentration of cellular markers (higher or lower than the median concentration) and by the expression of the mRNA of molecular markers (positive or negative) measured in the residual tumor.

Univariate and multivariate logistic regression analyses were conducted to estimate the variables associated with the lack of response to NAC in terms of odd ratios (ORs) and their associated 95% confidence intervals (CIs). Markers with a significance of *p* < 0.10 in the univariate regression model were evaluated in the final multivariate models to study effects and interactions of covariates on the outcome. The final model included all variables with a level of significance of *p* < 0.05, thereby creating a predictive model for patients not responding to NAC. The model obtained was internally validated through the bootstrapping simulation technique using IBM SPSS Statistics for Windows version 23.0 (IBM Corp., Armonk, NY, USA). Means and 95% Cis were estimated from the 10,000 bootstrapped data sets. The ability of the final multivariate regression models to predict the likelihood of failing to respond to NAC before and after bootstrapping validation was assessed by considering the sensitivity, specificity and area under the curve (AUC) of the receiver operating characteristic (ROC) curves. Statistical analyses and K-M curves were performed with IBM SPSS for Windows version 21.0 (IBM Corp., Armonk, NY, USA) and Stata software version 14.0 (StataCorp LLC, College Station, TX, USA), and graphical illustrations were generated with GraphPad Prism software (version 9.0, GraphPad Inc., San Diego, CA, USA).

## 3. Results

**Figure 1:**
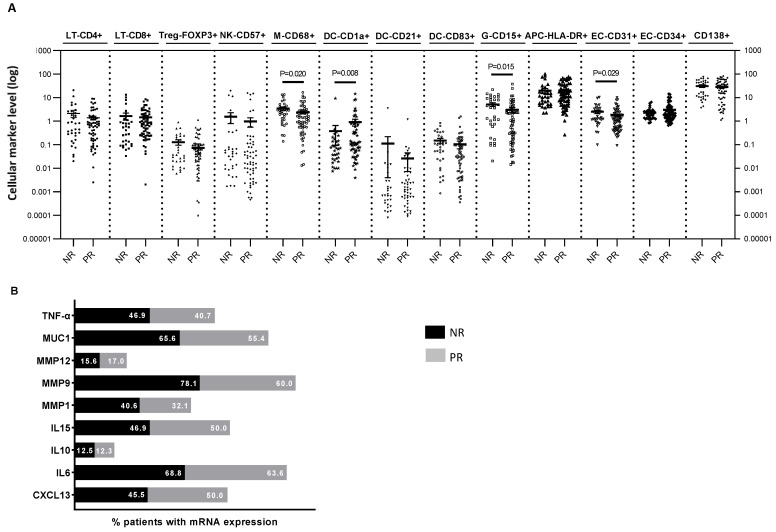
Immune cell content (**A**) and percentage of mRNA expression (**B**) determined in the residual tumors of TNBC patients. In (**A**), each dot represents the log-transformed (for ease of visualization) level of the immune markers of an individual patient. The horizontal line corresponds to the mean/95% confidence interval (CI) or the median/standard error of the median (SEM) depending on whether the values were normally distributed. Probabilities were those of the Mann–Whitney U test of the untransformed data. In (**B**), data are presented as the percentage of patients expressing mRNA in each category (NR vs. PR). NR: non-responders, PR: partial-responders, LT: T-lymphocytes, Treg: regulatory T cells, NK: natural killer cells, M: macrophage; DC: dendritic cells, G: granulocytes, APC: antigen-presenting cells, EC: endothelial cells. CXCL13: C-X-C motif chemokine ligand 13, IL: interleukin, MMP: matrix metalloproteases, MUC1: tumor-associated epithelial oncoprotein mucin-1, TNF-alpha: tumor necrosis factor alpha.

Compared with non-responders, partial responders had smaller tumors, a lower percentage of positive nodal status at diagnosis and longer OS (Supplementary Table S1). Regarding the cellular components in the residual TME, non-responder patients presented higher levels of CD68^+^ macrophages, CD15^+^ granulocytes and CD31^+^ endothelial cells, and a significantly lower level of Langerhans CD1a^+^ dendritic cells (DCs) than partial responders (Figure 1A). The mRNA expression of markers, quantified by in situ hybridization, was similar in the two groups (Figure 1B).

**Figure 2:**
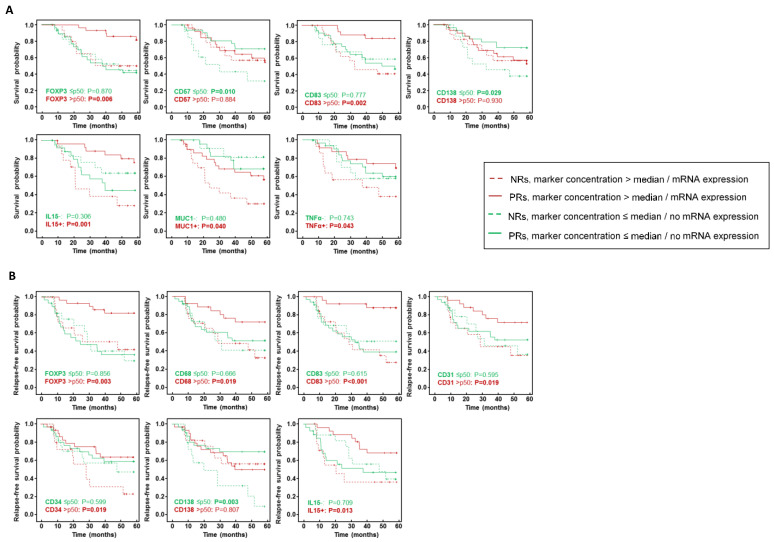
Superimposed Kaplan–Meier analysis of (**A**) five-year overall survival (OS) and (**B**) five-year relapse-free survival (RFS) with respect to significant type of response after neoadjuvant chemotherapy (NAC), stratified by concentration of cellular markers (greater than the median/less than or equal to the median) and absence/presence of mRNA expression of molecular markers. Significance of the log-rank test is indicated in the plots for each data set, with significant probabilities highlighted in bold. NR: non-responders, PR: partial responder.

To estimate whether the type of response is related to survival and relapse probability, we constructed Kaplan–Meier (K–M) curves using OS and RFS as endpoints. Although the OS and RFS were better in partial-responder patients than non-responders, K–M analysis found no significant association among the types of response (OS: *p* = 0.106; DFS: *p* = 0.068). Remarkably, stratifying patients by the concentration of cellular markers (greater than the median/less than or equal to the median) and the expression of molecular markers (presence or absence of mRNA expression) revealed significant differences (Figure 2). A high concentration of FOXP3^+^ T regulatory (Treg) cells, mature DCs (mDCs) CD83^+^ and IL-15, MUC1 and TNF-α mRNA expression but a low concentration of CD57^+^ natural killer (NK) and CD138^+^ plasma cells in partial responders yielded a better OS than in the non-responder group (Figure 2A). Compared with non-responders, patients with a partial response showed a significantly lower risk of relapse when their residual tumor exhibited high FOXP3, CD68, CD83, CD31 and CD34 content and IL15 expression but a low CD138 concentration (Figure 2B).

In evaluating variables associated with the type of response to NAC (Table 1), univariate regression determined large tumor diameter and positive nodal status at diagnosis to be factors associated with the lack of response to NAC (non-responder patients). Multivariate logistic regression found tumor diameter, positive nodal status at diagnosis and matrix metalloproteinase-9 (MMP-9) mRNA expression in the residual tumor to be factors associated with the absence of response to NAC.

**Figure 3:**
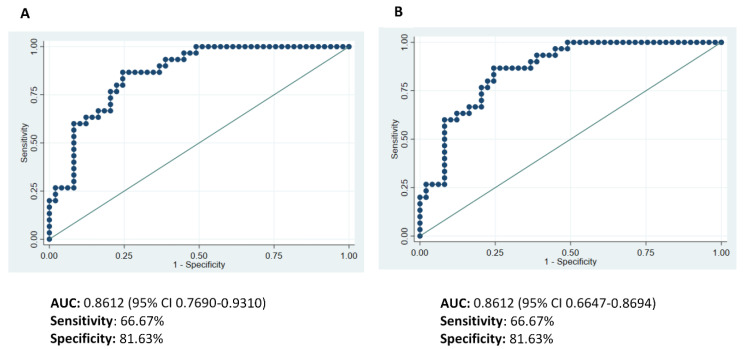
Receiver operating characteristic (ROC) curves evaluating the capacity of the final multivariate model to differentiate PR from NR patients with respect to the response to neoadjuvant chemotherapy (NAC): (**A**) before and (**B**) after bootstrapping.

The ability of the multivariate regression model to predict the likelihood of partial response or non-response to NAC was assessed by considering the AUC of the ROC curves (Figure 3). The multivariate models obtained before (Figure 3A) and after bootstrapping validation (Figure 3B) produced identical AUCs, sensitivities and specificities, only slightly extending the confidence interval after bootstrapping validation.

## 4. Discussion

Although NAC has substantially changed the TNBC paradigm, only patients who develop a pCR will clinically benefit from it [5,18]. Thus, effective biomarkers predicting the type of response after NAC are necessary to improve therapeutic strategies for non-responders. In our study, MMP-9 expression along with positive nodal status and large tumor size proved to be independent factors associated with the lack of pathological response to NAC (non-responders) compared with partial responders. As recently noted, although clinico-pathological factors are potent prognostic markers, they are not sufficiently robust to solely predict pCR rates and thereby correctly stratify TNBC patients [19], so other TME markers, such as MMP-9, must be considered. MMP-9, the most widely investigated protease in carcinogenetic processes, degrades the extracellular matrix, leading to the migration and invasion of cancerous cells, tumor metastasis, angiogenesis and inflammation [20]. Consistent with our findings, a high level of MMP-9 expression was correlated with risk of relapse or death in non-responders [21] and associated with poor BC-specific survival rates [22] and shorter OS [23]. Moreover, its overexpression correlates with lymph node metastasis and larger tumors [23]. In our cohort, there is a tendency towards a higher level of expression of MMP-9 in non-responders than in partial responders, even though the differences between the groups were not statistically significant. Interestingly, upregulation of not only MMP-9 but also other MMPs such as MMP-1, -7, -11, -13 and -14, proved to be predictive factors of poor prognosis in the serum of BC patients [24,25]. This is further evidence of the potential value of MMP members as biomarkers for predicting BC patient outcomes.

The fact that MMP-9 turned out to be an independent factor for non-response in the multivariate but not in the univariate model could be attributed to the presence of interactions between the predictors that only become apparent when considering the other covariates. Moreover, the degree of similarity between the two patient groups, whereby neither achieves pCR, might also account for this “masking” effect. Indeed, the difference between our study and most others lies in the fact that they compare patients with and without pCR (including partial responders and non-responders together). Nonetheless, K–M curves showed the differential effect of the concentration of some markers on OS and RFS probabilities on partial responders. The role of DC CD83^+^ in the response to NAC has hitherto been poorly investigated, and one of the few studies addressing this cell subset was performed in circulating cells with different post-NAC responses [26]. Dual function of FOXP3^+^ Treg cells in residual TME is elusive, especially in the neoadjuvant setting. While 40% achievement of pCR in a TNBC patient cohort was primarily attributed to the high level of TIL expression and the absence of FOXP3^+^ Treg cells [27], our study found improved OS and RFS in partial responders containing high levels of FOXP3^+^ Treg cells in the TME. In any case, as previously noted [12], increased FOXP3^+^ Treg cell levels after NAC could be a surrogate marker of improved survival and RFS in TNBC but would not be that useful in predicting the type of response to treatment in the post-NAC setting. Considering the last significant cellular marker for both OS and RFS, several works reported the involvement of CD138^+^ cells in pathological processes such as angiogenesis, cell proliferation, dissemination and cell migration. In accordance with our findings, the elevated expression of CD138 was associated with poor outcomes and aggressive BC phenotypes [28]. Regarding molecular markers, IL-15 is known to act as an immune-enhancing cytokine that is crucial for the survival, proliferation and functions of NK, T and B cells, which exhibited in vitro anti-tumor activity in TNBCs [29]. However, its prognostic value or relation to the response to NAC in TNBC patients remains unexplored. Further studies are therefore needed to identify the most powerful TME biomarkers for predicting the likelihood of response to NAC.

The crucial role of residual TME elements in the response to NAC and patient evolution is increasingly evident [12,30]. Our results reaffirm the suitability of combining cellular and molecular markers with classical clinico-pathological factors to distinguish early which TNBC patients will obtain the least clinical benefit from NAC so they can avoid unnecessary toxicity and allow the use of adjuvant therapies after surgical intervention to be delayed or even omit the surgery, thereby ultimately optimizing the schedule of clinical procedures. Additionally, standardizing the methods used to measure MMP-9 could facilitate its integration into clinical BC management. We believe this approach provides valuable knowledge about the mechanistic pathways in addition to the distinct response rates to treatments and establishes new therapeutic targets for post-NAC treatment development.

## Figures and Tables

**Table 1:** Univariate and multivariate logistic regression analysis of clinic-pathological factors and cellular markers associated with the absence of response to NAC in residual tumors of TBNC patients.

	Univariate Analysis		Multivariate Analysis		Multivariate Analysis after Bootstrapping	
	OR (95% CI)	*p*	OR (95% CI)	*p*	OR (95% CI)	*p*
Tumor diameter	1.032 (1.009–1.056)	**0.006**	1.041 (1.011–1.072)	**0.007**	1.041 (1.011–1.072)	**0.003**
Node status Positive Negative	11.177 (3.487–35.825) 1.0	**<0.001**	10.073 (2.650–38.280) 1.0	**0.001**	10.073 (2.650–38.280) 1.0	**<0.001**
MMMP9 Presence Absence	2.381 (0.879–6.451) 1.0	0.088	7.391 (1.818–30.052) 1.0	**0.005**	7.391 (1.818–30.052) 1.0	**0.003**

## Data Availability

The datasets used and/or analyzed during the current study are available from the corresponding authors on reasonable request.

## Supplementary Materials

The following supporting information can be downloaded at https://www.mdpi.com/article/10.3390/ijms241411297/s1.
