# Supplementary material for: Matrix Metalloproteinase-9 Expression Is Associated with the Absence of Response to Neoadjuvant Chemotherapy in Triple-Negative Breast Cancer Patients
Source: Int J Mol Sci. 2023 Jul 11;24(14):11297. doi: 10.3390/ijms241411297 (PMC10378773; doi:10.3390/ijms241411297)
Supplement: Supplementary file 1 [file ijms-24-11297-s001.zip › ijms-2488859-supplementary.pdf]

Supplementary Table S1. Clinico-pathological characteristics of the cohort of 96 triple-negative breast cancer (TNBC) patients by type of response to neoadjuvant chemotherapy (NAC).

	**Partial responders (n=63)**	**Non-responders (n=33)**	***p***
Age Years, median (IQR)	51.00 (25.00)	55.00 (25.00)	0.452*
**Menopausal status**			
Pre-menopausal	24 (47.1%)	11 (40.7%)	0.768†
Menopausal	27 (52.9%)	16 (59.3%)	
**Histological grade**			
< 3	10 (16.7%)	7 (21.9%)	0.741†
3	50 (83.3%)	25 (78.1%)	
**Tumour diameter**			
mm, median (IQR)	22.00 (21.00)	30.00 (35.00)	**0.003***
**Nodal status**			
Negative	37 (60.7%)	4 (12.1%)	**<0.001**†
Positive	24 (39.3%)	29 (87.9%)	
**Ki67 degree**			
≤ 30%	14 (25.9%)	10 (31.3%)	0.777†
> 30%	40 (74.1%)	22 (68.8%)	
**Surgery**			
Tumorectomy	26 (42.6%)	10 (30.3%)	0.342†
Mastectomy	35 (57.4%)	23 (69.7%)	
No lymphadenectomy	17 (27.9%)	3 (9.1%)	0.063†
Lymphadenectomy	44 (72.1%)	30 (90.9%)	
**Adjuvant treatment**			
No	51 (83.6%)	26 (81.3%)	1.000†
Yes	10 (16.4%)	6 (18.8%)	
**Radiotherapy**			
No	4 (4.6%)	0 (0.0%)	0.295†
Yes	54 (93.1%)	30 (100.0%)	
**Relapse**			
No	40 (63.5%)	14 (43.8%)	0.106†
Yes	23 (36.5%)	18 (56.3%)	
**Overall survival** (median months (IQR))	59.80 (34.93)	38.37 (36.17)	**0.026***
**Relapse-free survival** (median months (IQR))	45.47 (45.80)	29.53 (40.13)	0.056*
**Status**			
Alive	42 (66.7%)	17 (51.5%)	0.219†
Dead	21 (33.3%)	16 (48.5%)	

Data are presented as the median and interquartile range (Mann–Whitney U test, *), and the number and percentage of patients in each category (chi-squared test, †). Age, menopausal status, histological grade, tumour diameter, nodal status, and Ki67 degree were recorded at the time of diagnosis. Overall survival and relapse-free survival were censored at 60 months.
